# A Novel ERF Transcription Factor, *ZmERF105*, Positively Regulates Maize Resistance to *Exserohilum turcicum*

**DOI:** 10.3389/fpls.2020.00850

**Published:** 2020-06-16

**Authors:** Zhenyuan Zang, Ying Lv, Shuang Liu, Wei Yang, Jiabin Ci, Xuejiao Ren, Zhen Wang, Hao Wu, Wenyu Ma, Liangyu Jiang, Weiguang Yang

**Affiliations:** ^1^College of Agriculture, Jilin Agricultural University, Changchun, China; ^2^Crop Science Post-doctoral Station, Jilin Agricultural University, Changchun, China

**Keywords:** maize, *E*. *turcicum*, ethylene response factor, *ZmERF105*, transcription factor

## Abstract

The ethylene response factor (ERF) plays a crucial role in plant innate immunity. However, the molecular function of ERF in response to *Exserohilum turcicum* (*E*. *turcicum*) remains unknown in maize. In this study, a novel ERF gene, designated as *ZmERF105*, was firstly isolated and characterized. The *ZmERF105* protein contains an APETALA2/ETHYLENE RESPONSIVE FACTOR (AP2/ERF) domain and a conserved LSPLSPHP motif in its C-terminal region. ZmERF105 protein was exclusively localized to the nucleus. *ZmERF105* expression responded to *E*. *turcicum* treatment. Yeast one-hybrid and transcription activity assays revealed that *ZmERF105* is an activator of transcription and binds to GCC-box elements. Over-expression of *ZmERF105* was shown to increase maize resistance against *E*. *turcicum*, and *erf105* mutant lines displayed opposite phenotype. Moreover, the activities of superoxide dismutase (SOD) and peroxidase (POD) in the *ZmERF105* over-expression lines were markedly higher than in the wild-type maize lines (WT) after infection with *E*. *turcicum*, and were compromised in the *erf105* mutant lines. Simultaneously, *ZmERF105* over-expression lines enhanced the expression of several pathogenesis-related (PR) genes, including *ZmPR1a*, *ZmPR2*, *ZmPR5*, *ZmPR10.1*, and *ZmPR10.2* after infection with *E*. *turcicum*. In contrast, the expression of PR genes was reduced in *erf105* mutant lines. Our work reveals that *ZmERF105* as a novel player of the ERF network and positively regulates the maize resistance response to *E*. *turcicum*.

## Introduction

Maize *(Zea mays L*.) is one of the most important food crops in the world. Its productivity is frequently hampered by pathogens and so improved disease resistance is an important goal in many breeding projects (Galiano-Carneiro and Miedaner, 2017; Weems and Bradley, 2018). Northern corn leaf blight (NCLB), caused by *Exserohilum turcicum* (*E. turcicum*), is a destructive disease of maize worldwide (Chang and Fan, 1986). It can reduce crop yields approximately 50%, severe infection even results in a total yield loss (Raymundo and Hooker, 1981; Perkins and Pedersen, 1987). Resistant cultivars are the most effective and economical way of controlling NCLB. Nine qualitative *Ht* genes which confer resistance to specific races of *E. turcicum* have already been identified in maize, including *Ht1*, *Ht2*, *Ht3*, *HtN*, *Htm1*, *Htn1*, *HtP*, *ht4*, and *rt* (Carson, 1995; Welz and Geiger, 2000; Hurni et al., 2015). Qualitative resistance often results in breakdown of disease resistance because of a change in the pathogen population; quantitative resistance breeding has become the primary method for NCLB control (Carson, 1995; Welz and Geiger, 2000). Thus, it is necessary to isolate resistance genes and analyze their funtions in order to improve maize resistance to *E. turcicum*.

Plants have evolved an array of defense mechanisms that enable them to protect themselves from pathogen infection (Boller and He, 2009; Cui H. T. et al., 2009; Bigeard et al., 2015; Birkenbihl et al., 2017). APETALA2/ETHYLENE RESPONSIVE FACTOR (AP2/ERF) are thought to be the major transcription factors (TFs) that regulate the plant defense system (Nakano et al., 2006; Li et al., 2011; Pieterse et al., 2012; Lai et al., 2013; Lu et al., 2013; Sherif et al., 2013). They were usually classified to three families, including AP2, RAV, and ERF family (Licausi et al., 2013). The ERF family is sometimes further divided into two major subfamilies, the ERF subfamily and the DREB subfamily (Sakuma et al., 2002). The ERFs contain a highly conserved AP2/ERF domain consisting of 57 or 59 amino acid residues, and the AP2/ERF domain binds to the *cis*-acting element AGCCGCC (GCC-box) presents in the promoters of pathogenesis-related (PR) genes (Ohme-Takagi and Shinshi, 1995; Zarei et al., 2011; Cheng et al., 2013; Franco-Zorrilla et al., 2014). The ERF gene was firstly isolated in tobacco (Ohme-Takagi and Shinshi, 1995), and successively identified in other plant species, such as wheat (Xu et al., 2007), tomato (Pan et al., 2010), soybean (Zhai et al., 2013) and rice (Zhang et al., 2013).

ERF genes acting as transcription activators or repressors involved in plant defense reactions (McGrath et al., 2005; Sherif et al., 2013; Huang et al., 2016; Li et al., 2018; Huang et al., 2019). Over-expression of *AtERF1* activated the expression of PLANT DEFENSINS (*PDF1.2*) directly, and increased the plants resistance against a range of pathogens (Berrocal-Lobo et al., 2002; Lorenzo et al., 2003). A T-DNA insertion mutant of *AtERF14* increased susceptibility to infection by *Fusarium oxysporum* (*F. oxysporum*) (Oñate-Sánchez et al., 2007)*. AtERF96* positively regulated the *Arabidopsis* resistance to necrotrophic pathogen through enhancing the expression of *PDF1.2a*, *PR-3*, *PR-4*, and *ORA59* (Catinot et al., 2015). The transcription activator, *AtERF15* could positively regulate the *Arabidopsis* resistance against *Pseudomonas syringae pv. tomato* (*Pst*) *DC3000* and *Botrytis cinerea* (*B. cinerea*) (Zhang et al., 2015). Although many ERF TFs identified so far are transcription activators, several ERFs were shown to act as negatively regulators of plant defense mechanisms (McGrath et al., 2005; Maruyama et al., 2013; Tian et al., 2015). Over-expression of *AtERF4* was more susceptible to *F. oxysporum*, and knockout mutants of *AtERF9* showed enhanced resistance to *B. cinerea* by suppressing the expression of the *PDF1.2* (McGrath et al., 2005; Maruyama et al., 2013). *StERF3*, which contains an EAR motif, negatively regulates resistance to *Phytophthora infestans* (*P. infestans*) (Tian et al., 2015).

Previous reports have demonstrated that many ERF genes respond to various stimulus, such as ethylene (ET), salicylic acid (SA), and jasmonic acid (MeJA) (Grant and Jones, 2009; Cheng et al., 2013; Müller and Munné-Bosch, 2015; Yang et al., 2015; Zhang et al., 2017; Xie et al., 2019). Transcription of the *TaERF1* is induced by ABA, ET, and SA (Xu et al., 2007). Expression of *GmERF7* is induced by treatment with MeJA, ET, and ABA (Zhai et al., 2013). Transcription of the *GmERF113* can be induced by ABA, ET, and SA (Zhao et al., 2017). These stimulus have been associated with resistance against different types of pathogens, such as (hemi) biotrophic pathogens are modulated *via* SA signaling pathway while necrotrophic pathogens are mediated through MeJA/ET signaling pathway (Ward et al., 1991; Farmer et al., 2003; Yang et al., 2005; Vlot et al., 2009; Verhage et al., 2010; Caarls et al., 2017). For example, *AtERF1*, *AtERF5*, *AtERF6*, and *ORA59* are suggested to participate in JA/ET-regulated defense responses and increased plants resistance against *B. cinerea* by promoting the expression of *AtPDF1.2* and *ChiB* (Lorenzo et al., 2003; Pre et al., 2008; Leon-Reyes et al., 2010; Zarei et al., 2011; Son et al., 2012; Moffat et al., 2012; Cheng et al., 2013; Van der Does et al., 2013; He et al., 2017; Kim et al., 2018). *ERF11* acted as a regulator of the SA-mediated signaling pathway to enhance the *Arabidopsis* defense response against *Pst DC3000* by directly regulating the expression of *BT4* (Zheng et al., 2019).

Although the regulatory functions of the ERF genes have been widely explored in plants, the role of ERF genes in response to *E*. *turcicum* remain unknown. In a previous study, a novel ERF gene was significantly induced after infection with *E. turcicum* in the resistant inbred line “Mo17” than in the susceptible inbred line “Huobai”. In this work, we firstly isolated and characterized this ERF gene in maize, designated as *ZmERF105* (GenBank No. NM001177195). ZmERF105 protein was exclusively localized to the nucleus. ZmERF105 acts as a transcriptional activator that binds to GCC-box elements. *ZmERF105* expression responded to *E*. *turcicum* treatment. *ZmERF105* positively regulated plant resistance against *E. turcicum via* regulating the expression of defense-related genes and the activities of antioxidant enzymes. These data suggested that *ZmERF105* plays an important role in plant defense reactions, and may be useful in molecular breeding to improve the defensive capacity of maize against *E*. *turcicum*.

## Materials and Methods

### Plant Materials and Stress Treatments

*E. turcicum* and maize inbred lines were provided by Maize Breeding Team in Jilin Agricultural University. The seeds of the maize inbred line B73 were germinated in a greenhouse under a long-day photoperiod (16-h light, 8-h dark), and watered once every 3 days. Plant stress treatments were performed at the three-leaf stage. For hormone treatments, maize seedlings were sprayed with 0.1 mM MeJA, 0.1 mM ABA, or 0.5 mM SA. ET treatment was performed in sealed plexiglass chamber by dissolving 2 ml of 40% ethephon and 1 g of NaHCO_3_ in 200 ml of H_2_O.

*E. turcicum* (mixed races) was inoculated into the maize inbred line Mo17 (resistant inbred line) and Huobai (susceptible inbred line) according to the methods described by Wu et al. (2014), The plants were inoculated with two to three drops of conidial suspension at six-leaf stage, and the concentration of conidia was estimated to be about 1 × 10^5^ spores ml^−1^. After inoculation, the plants were kept at 100% relative humidity to ensure spore germination. The leaves were collected at 6, 12, 24, 48, and 72 h after the treatment, and were frozen in liquid nitrogen for RNA extraction.

### Cloning of *ZmERF105* and Phylogenetic Analysis

The full-length cDNA sequence of *ZmERF105* was amplified from the leaves of the maize inbred line B73 using reverse transcription PCR (RT-PCR). The amplified products were cloned into the pMD-18T vector (Takara) for sequencing (Sangon, Shanghai, China). The amino acid sequences of *ZmERF105* and other AP2/ERF members were obtained from NCBI database (https://blast.ncbi.nlm.nih.gov/Blast.cgi). Multiple sequence alignment was analyzed using DNAMAN software, and the phylogenetic tree was generated with the neighbor-joining method using MEGA 5.0 software. The primers used for *ZmERF105* cloning were provided in **Supplementary Table S1**.

### Quantitative Real-Time PCR Analysis

Total RNA was extracted from maize leaves using Trizol reagent (Invitrogen, China) and reverse transcription was performed by using 1 µg of total RNA. Quantitative real-time PCR (qRT-PCR) was performed on a QuantStudio 3 instrument (Thermo, USA) using a SYBR Mixture system (TOYOBO, Japan). The relative expression levels were calculated by the 2^−ΔΔCt^ method with the maize housekeeping gene *ZmTub* (GRMZM2G066191). The expression analysis was performed using three biological and three technical replicates, and statistically analyzed using Student’s t-test (*P < 0.05, **P < 0.01). The primers used for expression analysis were provided in **Supplementary Table S1**.

### Maize Transformation and Assessment of Plant Disease Resistance

The coding sequence of *ZmERF105* was ligated into the *Nc*oI and *Pm*lI sites of pCAMBIA3301 vector to construct the CaMV 35s: *ZmERF105* plasmid, and the recombinant plasmid was introduced into *Agrobacterium tumefaciens* strain LBA4404 using the freeze-thaw method (Holsters et al., 1978). Germinating embryos of the maize inbred line H99 (susceptible inbred line) were used as explants transformed with the *Agrobacterium*-mediated method (Zhou et al., 2011). T_2_ transgenic maize lines were further confirmed using PCR and qRT-PCR. Primers used for the vector construction and detection were presented in **Supplementary Table S1**.

*erf105* UFMu mutant lines (W22, mu2041934) were obtained from the Maize Genetics Cooperation Stock Center. The homozygous mutant lines were identified using PCR. The transcription levels were detected using qRT-PCR as described previously. Primers used for detection were presented in **Supplementary Table S1**.

To investigate the transgenic and mutant maize lines resistance against *E. turcicum*, artificial inoculation procedures were performed as described by Wu et al. (2014). The wild-type maize lines (WT) were used as the control. The living ear leaves of T_2_ transgenic lines and mutant lines were infected with *E. turcicum*. Disease symptoms on infected leaves were observed with a Nikon D7000 camera at 5 days post-inoculation (dpi). The relative lesion area was measured as described by Cui H. W. et al. (2009) using Photoshop CS3.

### Subcellular Localization of ZmERF105

The full-length *ZmERF105* sequence was cloned and ligated into the *Kp*nI and *Xb*aI sites of pCAMBIA1300 vector to construct the CaMV 35s: *ZmERF105-GFP* plasmid. After sequencing confirmation, CaMV 35s: *ZmERF105-GFP* plasmid and empty vector were transiently expressed in *N. benthamiana* (Liu et al., 2010). The GFP fluorescence signal was visualized and photographed with a laser scanning confocal microscope (Leica TCS SP5, Germany). The primers used for expression analysis were provided in **Supplementary Table S1**.

### Yeast One-Hybrid Assay

The coding sequence of *ZmERF105* was amplified and ligated into the *Nc*oI and *Eco*RI sites of the pGADT7 vector. The specific DNA fragments, GCC (ATCCATAAGAGCCGCCACTAAAATAAGACCGATCAA) and mutated GCC (mGCC) (ATCCATAAGATCCTCCACTAAAATAAGACCGATCAA) were inserted into the pHIS2 vector. The plasmids were transformed into yeast strain Y187 and the transformants were selected on SD (-Trp/-Leu). Transformed colonies were subsequently grown on SD (-Trp/-His/Leu) medium with 100 mM 3-amino-1, 2, 4-triazole (3-AT), and were cultured at 28°C for 3 days. Primers used for the vector construction were presented in **Supplementary Table S1**.

### Transcription Activity Assay

For transcription activity assay, the coding sequence of *ZmERF105* was inserted into *Nc*oI and *Pm*lI sites of pCAMBIA3301 vector to construct the CaMV 35s: *ZmERF105* effector plasmid. The reporter plasmid was constructed according to the method described by Dong et al. (2015). The combined reporter and effector plasmids were cotransformed into the *Arabidopsis* protoplasts according to a protocol described by Yoo et al. (2007). The relative GUS activity was determined as described by Chen et al. (2006). Primers used for the vector construction were presented in **Supplementary Table S1**.

### Enzyme Activity Assay

The fresh leaves (approximately, 0.1 g) of seedlings were harvested and homogenized in 1 ml of 50 mM ice-cold phosphate buffer (pH 7.8). The extract was centrifuged at 12,000 rpm for 20 min at 4°C. Superoxide dismutase (SOD) and Peroxidase (POD) activities were measured following the method that described by Li et al. (2015).

## Results

### Isolation and Sequence Analysis of ZmERF105

**Figure 1:**
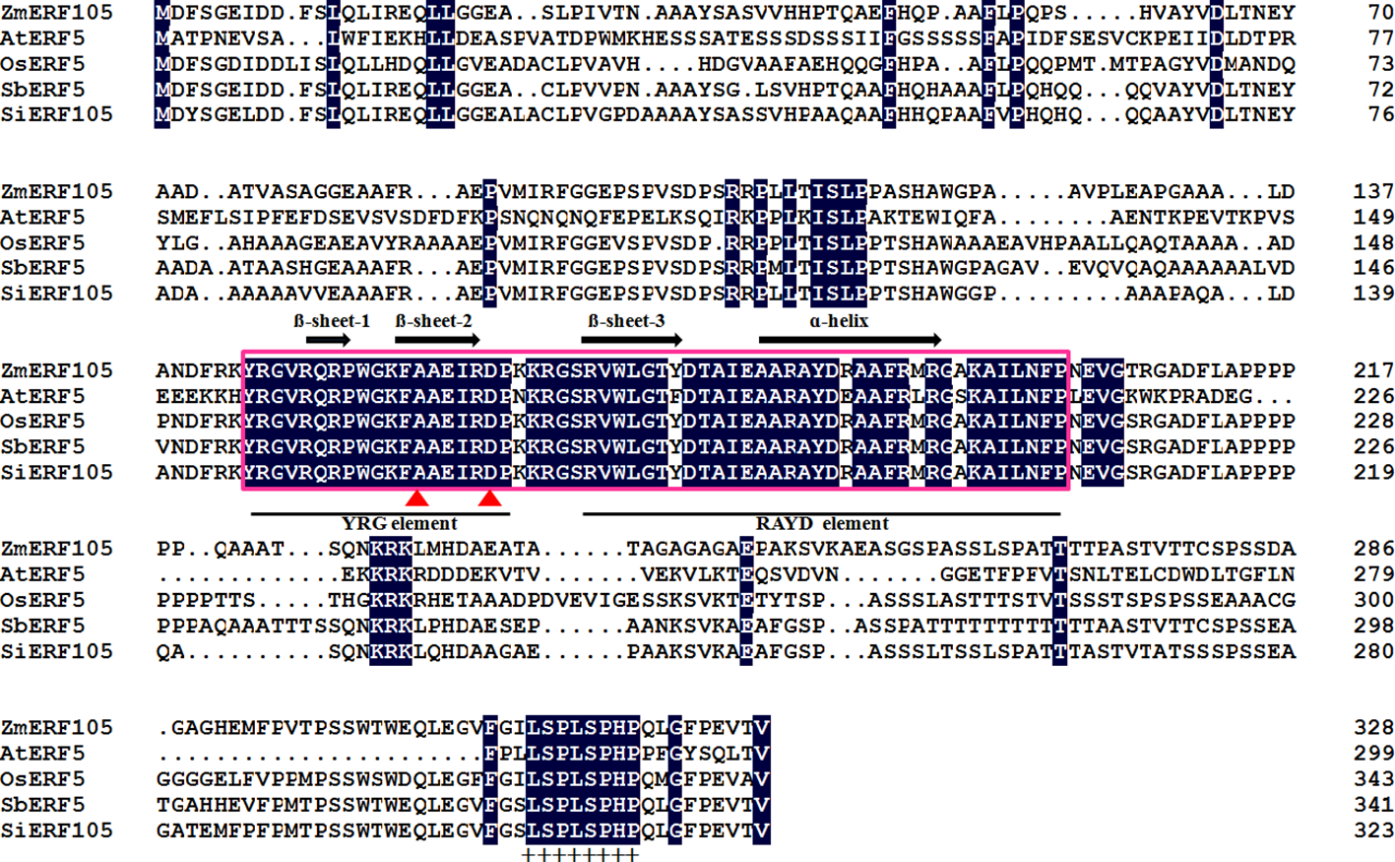
Conserved motifs of the ZmERF105 protein sequence in alignment with other ERF transcription factors. The sequence alignment was performed using DNAMAN software. The AP2 DNA binding domain is indicated by pink box. The one α-helix and three β-sheets are marked above the corresponding sequences. The YRG and RAYD elements are indicated below the alignment. The conserved alanine and aspartic acid residues in the AP2 domain are marked by red triangles. A conserved LSPLSPHP motif is marked by ‘‘+’’. AtERF5 (NP568679) is derived from *Arabidopsis thaliana*, OsERF5 (XP015624058) is derived from *Oryza sativa*, SbERF5 (XP021315889) is derived from *Sorghum bicolor*, and SiERF105 (XP004953326) is derived from *Setaria italica*.

**Figure 2:**
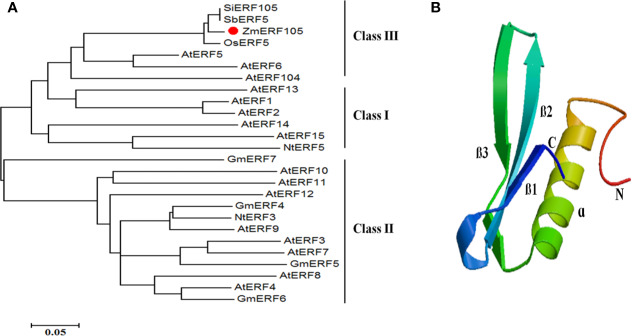
The phylogenetic tree and predicted three-dimensional structure of ZmERF105 protein. **(A)** The phylogenetic tree was generated with the neighbor-joining method using MEGA 5.0 software. ZmERF105 is indicated by the red dot. The accession numbers are as follows: AtERF1 (NP188965), AtERF2 (NP199533), AtERF3 (NP175479), AtERF4 (NP188139), AtERF5 (NP568679), AtERF6 (NP567529), AtERF7 (NP188666), AtERF8 (NP175725), AtERF9 (NP199234), AtERF10 (NP171876), AtERF11 (NP174159), AtERF12 (NP174158), AtERF13 (NP182011), AtERF14 (NP171932), AtERF15 (NP9850162), AtERF104 (NP_200968), GmERF4 (ACE76905), GmERF5 (AEX25891), GmERF6 (AEQ55267), GmERF7 (AEQ55266), NtERF3 (BAJ72664), NtERF5 (AAU81956), SbERF5 (XP021315889), SiERF105 (XP004953326), and OsERF5 (XP015624058). **(B)** Predicted three-dimensional structure of ZmERF105 protein.

The full-length cDNA sequence of *ZmERF105* (GenBank No. NM001177195) was isolated from the leaves of maize inbred line B73 using RT-PCR. *ZmERF105* has an open reading frame (ORF) of 990 bp, encoding a polypeptide of 329 amino acids with a predicted molecular mass of 34.5 kDa and a theoretical PI of 5.77. The deduced ZmERF105 protein contains a conserved AP2/ERF domain consisting of 58 amino acids, with conserved alanine (A) and aspartic acid (D) in it, suggesting that it is a member of the ERF family (**Figure 1**). The AP2/ERF domain contains conserved YRG and RAYD elements, which may play a key role in DNA binding and protein interaction, respectively (**Figure 1**). In addition, the ZmERF105 protein contains a conserved LSPLSPHP motif in its C-terminal region which is a mitogen-activated protein kinase (MAPK) phosphorylation site (**Figure 1**). The results from searching the Phytozome database (http://www.phytozome.net/maize) showed that *ZmERF105* did not have intron and is located on chromosome 5. The phylogenetic tree analysis indicated that ZmERF105 protein shows highly similarity to SiERF105 (GenBank No. XP004953326), SbERF5 (GenBank No. XP021315889), and OsERF5 (GenBank No. XP015624058), respectively (**Figure 2A**). According to Fujimoto et al. (2000), ZmERF105 protein belongs to class III ERF group. The results from searching the SWISS-MODEL database (https://swissmodel.expasy.org/) showed that the 3D structure of ZmERF105 has a long C-terminal α-helix (α) wrapped in a three-stranded anti-parallel β-sheet (β1–β3) (**Figure 2B**).

### Expression Profiles of ZmERF105 Under Various Stresses

**Figure 3:**
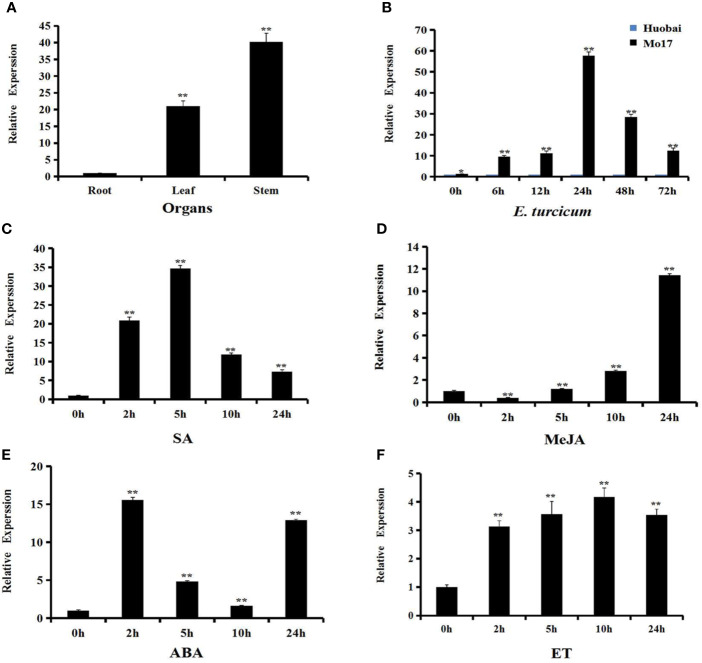
Expression profiles of *ZmERF105* in maize. **(A)** Expression analysis of *ZmERF105* in different organs. **(B)** Relative expression levels of *ZmERF105* in Mo17 (resistant inbred line) and Huobai (susceptible inbred line) plants infection with *E. turcicum*. The samples were collected at 0, 6, 12, 24, 48, and 72 h after *E. turcicum* infection. The relative expression levels were compared with susceptible inbred line Huobai at same points. **(C–F)** Expression analysis of *ZmERF105* under hormone treatments, including 0.5mM SA, 0.1mM MeJA, 0.1mM ABA or ET. The relative expression levels were calculated by the 2^-ΔΔCt^ method with the maize housekeeping gene *ZmTub* (GRMZM2G066191) as an internal control. The experiment was performed using three biological and technical replicates each, and analyzed using Student' *t*-tests (*P < 0.05, **P < 0.01). Bars indicate standard error of the mean (SE).

In order to better understand the function of *ZmERF105*, the expression levels of *ZmERF10*5 were investigated using qRT-PCR. The results demonstrated that *ZmERF10*5 was expressed, with the highest expression in stems, followed by the leaves and the roots (**Figure 3A**). Under SA and ET treatments, the expression levels of *ZmERF10*5 peaked at 5 and 10 h, respectively (**Figures 3C, F**). MeJA treatment induced a down-regulation of *ZmERF10*5 expression at 2 h, followed by a slow increase, with maximum at 24 h (**Figure 3D**). In contrast, *ZmERF10*5 expression rapidly reached the maximum at 2 h after ABA treatment, followed by a rapid decline in 5–10 h, and increased at 24 h (**Figure 3E**).

*ZmERF10*5 expression was detected in Mo17 (resistant inbred line) and Huobai (susceptible inbred line) plants after infection with *E. turcicum*. As shown in **Figure 3B**, the expression level of *ZmERF10*5 was higher in Mo17 leaves than that in Huobai leaves during *E. turcicum* infection. A significant up-regulation of *ZmERF10*5 expression was detected at 6 h and reached a maximum level at 24 h. These results demonstrated that *ZmERF10*5 may involve in response to pathogen and multiple stimulus.

### Subcellular Localization of the ZmERF105 Protein

**Figure 4:**
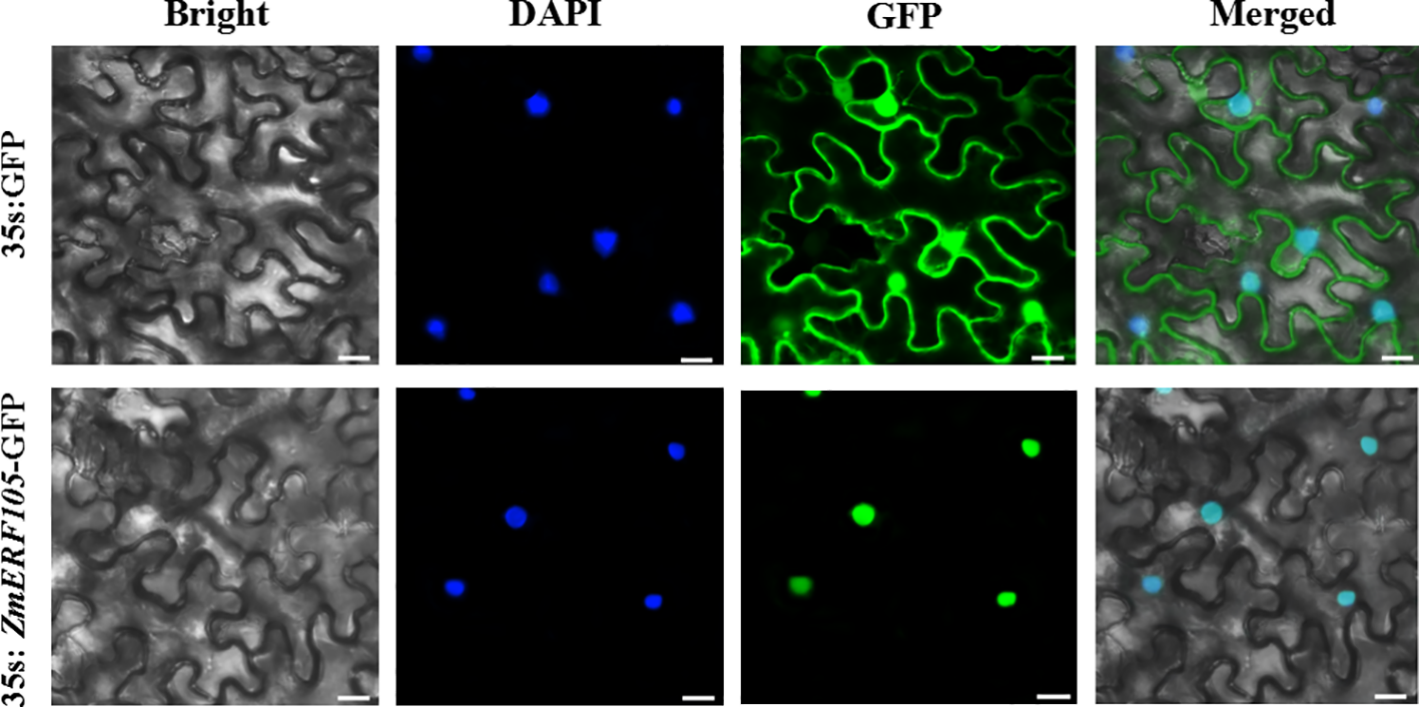
Subcellular localization of ZmERF105-GFP fusion protein in the epidermal cells of *N. benthamiana*. *N. benthamiana* leaves transiently expressing GFP alone (upper) and ZmERF105-GFP (bottom) proteins were observed with a confocal microscope. Bars = 25 μm.

To determine the subcellular location of ZmERF105 protein, a ZmERF105-GFP fusion protein under the control of the *35S* promoter was obtained and introduced into the epidermal cells of *N. benthamiana*. Consistent with its function as a TF, the ZmERF105-GFP fusion protein was exclusively localized to the nucleus, and the fluorescent signals of the control were observed in whole cells (**Figure 4**).

### ZmERF105 Binds to GCC-Box Elements and Functions as a Transcription Activator

**Figure 5:**
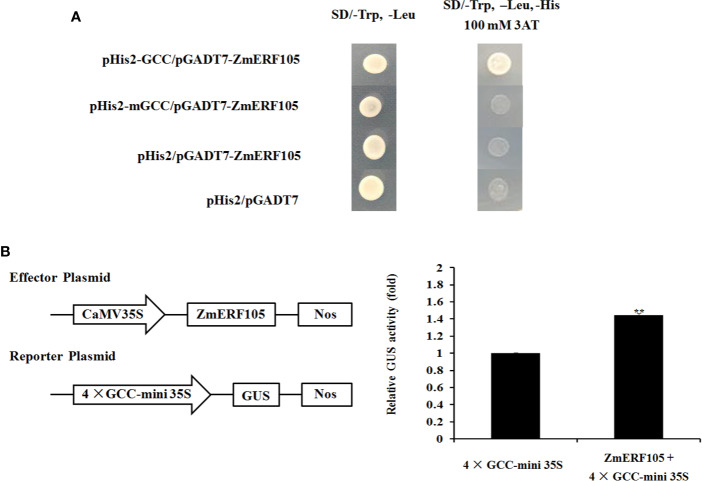
Binding to the GCC-box elements and transcription activity analysis of ZmERF105. **(A)** The binding activity of ZmERF105 to the GCC-box elements in yeast one-hybrid assay. Yeast cells were selected on SD (-Trp/-Leu) and SD (-Trp/-Leu/-His) media supplemented with 100 mM 3-AT. **(B)** Transcription activity analysis of ZmERF105 in *Arabidopsis* protoplasts. The numbers show the fold increase in GUS activity compared with the reporter vector driven by 4× GCC 35S mini promoter alone. The experiment was performed using three biological and technical replicates each and analyzed using Student’s *t*-tests (**P < 0.01). Bars indicate standard error of the mean (SE).

Previous studies have shown that ERF genes contain a conserved DNA-binding domain which can bind to GCC-box elements (Zhang et al., 2004; Tang et al., 2007). To further investigate whether ZmERF105 specifically binds to GCC-box elements, a yeast one-hybrid assay was performed. As shown in **Figure 5A**, the bait stain (pHIS2*-GCC*) transfected with prey vector (pGADT7*-ZmERF105*) could grow on SD (-Leu/-Trp/-His) medium containing 100 mM 3-AT, while mutant bait stain (pHIS2*-mGCC*) could not. The result indicated that ZmERF105 can bind specifically to GCC-box elements

Transient transcription activity assay was used to determine whether ZmERF105 functions as a transcription activator. Expression of ZmERF105 was driven by the CaMV 35S promoter, and the GUS reporter gene was driven by 4× GCC 35S mini promoter. As shown in **Figure 5B**, the relative GUS activity of the *Arabidopsis* protoplasts transformed with was the CaMV 35S: ZmERF105 and 4× GCC 35S: GUS was approximately 1.4 times as high as the control. These results demonstrated that ZmERF105 functions as a transcriptional activator and binds to GCC-box elements.

### ZmERF105 Positively Regulates Maize Resistance Against *E. turcicum*

**Figure 6:**
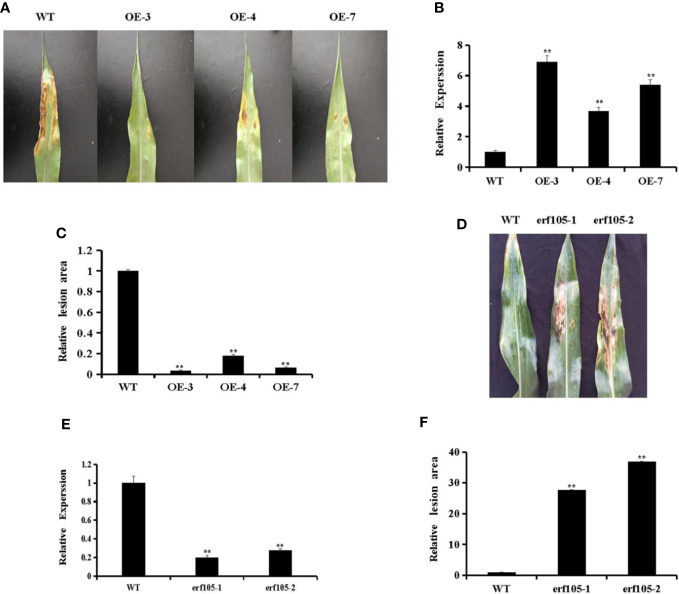
Responses of *ZmERF105* over-expression and *erf105* mutant lines to *E. turcicum*. **(A)** Disease symptoms on the leaves of T_2_ transgenic lines at 5 dpi. **(B)** Relative expression analysis of *ZmERF105* in T_2_ transgenic lines. **(C)** The relative lesion areas of the transgenic lines at 5 dpi. **(D)** Disease symptoms on the leaves of *erf105* mutant lines at 5 dpi. **(E)** Relative expression analysis of *ZmERF105* in *erf105* mutant lines. **(F)** The relative lesion areas of the *erf105* mutant lines at 5 dpi. The experiment was performed using three biological and technical replicates each and analyzed using Student’s *t*-tests (**P < 0.01). Bars indicate standard error of the mean (SE).

To investigate whether *ZmERF105* can improve resistance to *E. turcicum* in transgenic maize plants, T_2_ transgenic plants, constituting three independent *ZmERF105* over-expressing transgenic lines (OE3, OE4, and OE7) was used to investigate the response to *E. turcicum*. The expression of *ZmERF105* in the representative transgenic lines (OE3, OE4, and OE7) was further detected by qRT-PCR (**Figure 6B**). As illustrated in **Figures 6A, C**, the sizes of the lesions in over-expressing lines were significantly smaller than in the wild-type maize lines (WT) at 5 dpi. In addition, the relative expression of *ZmERF105* had about 0.2-fold and 0.3-fold declines in *erf105* mutant lines, respectively (**Figure 6E**). After inoculation with *E. turcicum*, the *erf105* mutant lines displayed significantly susceptibility compared with the WT at 5 dpi (**Figures 6D, F**). These results demonstrated that *ZmERF105* positively regulates the maize resistance against *E. turcicum*.

### ZmERF105 Affects Antioxidant Enzyme Activity

**Figure 7:**
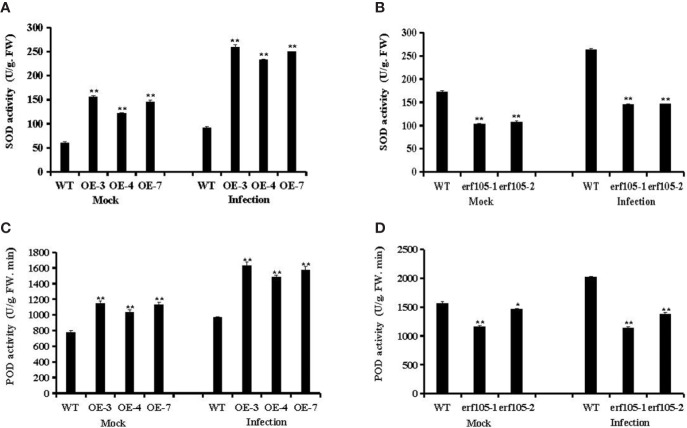
Analysis of antioxidant enzyme activity under mock treatment and infected by *E. turcicum* at 24-h post-inoculation (hpi). **(A, B)** The activity of the SOD in *ZmERF105* over-expression and *erf105* mutant lines, respectively. **(C, D)** The activity of the POD in *ZmERF105* over-expression and *erf105* mutant lines, respectively. The activity of the WT sample [mock-treated wild-type (WT) plants] was set to unity. The experiment was performed on three biological replicates, each with three technical replicates, and statistically analyzed using Student’s t-test (*P < 0.05, **P < 0.01). Bars indicate standard error of the mean (SE).

SOD and POD are the most important antioxidant enzyme in the process of ROS scavenging reaction, and it was suggested to participate in various defense mechanisms (Liu et al., 2016). To further confirm whether the role of *ZmERF105* in resistance against *E. turcicum* is related to SOD and PDD activities, we examined the activities of SOD and POD in *ZmERF105* over-expression and *erf105* mutant lines. After inoculation *E. turcicum*, both SOD and POD activities were significantly high in *ZmERF105* over-expression lines compared with those in the mock-inoculated lines at 24 h, and were remarkably decreased in the *erf105* mutant lines (**Figure 7**). These results suggested that *ZmERF105* increases the activities of the antioxidant enzymes in maize resistance to *E. turcicum*.

### ZmERF105 Involves in Various Plant Defense Responses

**Figure 8:**
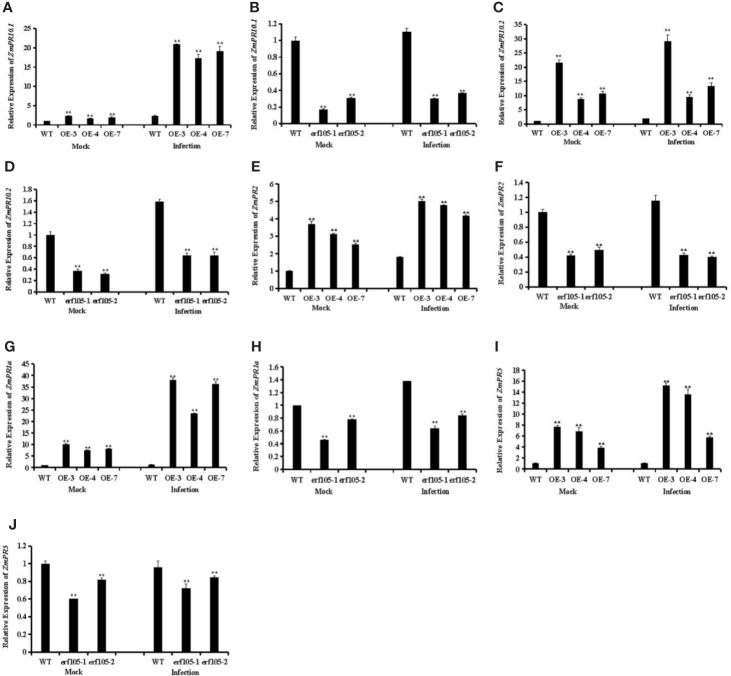
Relative expression levels of defense-related genes under mock treatment and infected by *E. turcicum* at 24 hpi in *ZmERF105* over-expression and *erf105* mutant lines, respectively. **(A, B)** The expression of the *ZmPR10.1*. **(C, D)** The expression of the *ZmPR10.2*. **(E, F)** The expression of the *ZmPR2*. **(G, F)** The expression of the *ZmPR1a*. **(H, I)** The expression of the *ZmPR5*. The relative expression levels were calculated by the 2^−ΔΔCt^ method with the maize housekeeping gene *ZmTub* (GRMZM2G066191) as an internal control. The expression of the WT sample [mock-treated wild-type (WT) plants] was set to unity. The experiment was performed using three biological and technical replicates each and analyzed using Student’s *t*-tests (**P < 0.01). Bars indicate standard error of the mean (SE).

ERF genes are suggested to participate in plant defense responses through regulating the expression of PR genes (Lorenzo et al., 2003; Pre et al., 2008; Moffat et al., 2012; Son et al., 2012). To further analyze the role of *ZmERF105* in resistance against *E. turcicum* in maize, we examined the expression of PR genes that including *ZmPR1a* (GRMZM2G465226), *ZmPR2* (GRMZM5G456997), *ZmPR5* (GRMZM2G402631), *ZmPR10.1* (GRMZM2G112488), and *ZmPR10.2* (GRMZM2G112538). As shown in **Figure 8**, after 24h incubation with *E. turcicum*, the expression levels of these PR genes were highly induced in *ZmERF105* over-expressing lines compared with those in the mock-inoculated lines, and were remarkably reduced in the *erf105* mutant lines. These results indicated that *ZmERF105* may enhance maize defense against *E. turcicum* by directly or indirectly regulating these PR genes.

Due to the expression levels of SA-associated genes *ZmPR1a* and *ZmPR5* exhibited significant increase in the *ZmERF105* over-expression lines and were decreased in the *erf105* mutant lines after *E. turcicum* infection, the expression level of *ZmERF105* was induced by SA treatment, we speculated whether *ZmERF105* is required for SA induced defense response. To test this hypothesis, we further assessed SA induces resistance to *E. turcicum* infection in *erf105* mutant lines. As shown in **Supplementary Figure S1**, *erf105* mutant lines showed less severe disease symptom by application of exogenous 10 mM SA than the water-treated lines at 5 dpi (**Supplementary Figure S1**). These results demonstrated that *ZmERF105* may involve in SA-induced defense response. To further confirm whether *ZmERF105* involved in MeJA, ET, and ABA induced defense responses, we detected the expression of MeJA-associated gene *ZmLox1* (GRMZM2G156861), ET-associated gene *ZmACS6* (GRMZM2G04361), and ABA-associated gene *ZmRD20* (GRMZM2G342685), respectively. The results showed that the expression levels of *ZmLox1* and *ZmACS6* significantly increased in the *ZmERF105* over-expression lines and were decreased in the *erf105* mutant lines after *E. turcicum* infection, while *ZmRD20* showed opposite results (**Supplementary Figure S2**). These results demonstrated that *ZmERF105* may act positively as regulators of MeJA and ET signaling pathways in resistance to *E. turcicum*, and negatively in ABA signaling pathway.

## Discussion

Plants have evolved an array of defense mechanisms to protect themselves when they are exposed to a wide variety of pathogens (Schwessinger and Ronald, 2012; Fu and Dong, 2013; Birkenbihl et al., 2017). ERFs are one of the most important TFs in the plant defense syetem (McGrath et al., 2005; Pre et al., 2008; Maruyama et al., 2013). In maize, a total of 98 predicted ERF members have been identified (Liu et al., 2013). In this study, a novel maize ERF gene, *ZmERF105*, was isolated and identified. *ZmERF105* is involved in the pathogen response pathway and positively regulates the maize resistance against *E. turcicum*.

To our knowledge, ERF families were divided into ERF subfamily and DREB subfamily according to the amino acids difference at position 14th and 19th in AP2/ERF domain (Sakuma et al., 2002). Similar to other ERF proteins, ZmERF105 protein contains a conserved AP2/ERF domain consisting of 58 amino acids, with conserved alanine (A) and aspartic acid (D) in it (**Figure 1**), suggesting that it is a member of the ERF subfamily. Sequence analysis showed that ZmERF105 protein contains a LSPLSPHP motif in its C-terminal region, which maybe indicate the role of ZmERF105 in MAPK signaling pathway (**Figure 1**).

ERF genes have been shown to act as transcription activators or repressors and are able to bind to GCC-box elements (Fujimoto et al., 2000). For example, AtERF1, ORA59, AtERF6, and AtERF96 serve as transcriptional activators while GmERF5 and StERF3 serve as transcriptional repressors, and they can bind to GCC-box elements (Zarei et al., 2011; Wang et al., 2013; Catinot et al., 2015; Dong et al., 2015; Tian et al., 2015). In this study, we found that nuclear loci of ZmERF105 binds specifically to the GCC-box elements by yeast one-hybrid assay and shows transactivation activity in *Arabidopsis* protoplast (**Figures 4** and **5**). Our results suggested that ZmERF105 may function as a transcription activator and regulate defense-related genes expression by binding to GCC-box elements present in their promoters.

Most of ERFs act as activators and positively regulate the plant resistance against pathogens (Berrocal-Lobo et al., 2002; Son et al., 2012). Over-expression of *AtERF1*, O*RA59*, *AtERF6*, *AtERF96*, and *AtERF104* were shown to increase plant resistance against *B. cinerea* (Berrocal-Lobo et al., 2002; Pre et al., 2008; Bethke et al., 2009; Zarei et al., 2011; Meng et al., 2013; Wang et al., 2013; Catinot et al., 2015). The transcription activator, *AtERF15* could positively regulate the *Arabidopsis* resistance against *Pst DC3000* and *B. cinerea* (Zhang et al., 2015). Over-expression of *GmERF113* in transgenic soybean led to increased resistance to *Phytophthora Sojae* (*P.sojae*) (Zhao et al., 2017). In our research, the expression of *ZmERF105* was significantly induced after infection with *E. turcicum* in the resistant inbred line “Mo17” than in the susceptible inbred line “Huobai” (**Figure 3B**). Therefore, we speculate that *ZmERF105* may play important role in response to *E. turcicum*. As a result, we further analyzed the function of *ZmERF105* in resistance to *E. turcicum*. Over-expression of *ZmERF105* was shown to increase maize resistance against *E*. *turcicum*, and *erf105* mutant lines displayed opposite phenotype (**Figure 6**). These results demonstrated that *ZmERF105* positively regulates the maize resistance response to *E*. *turcicum*.

ERF genes are mainly involved in plant defense response by directly regulating the expression of defense-related genes (Meng et al., 2013; Wang et al., 2013). *AtERF96* positively regulates the *Arabidopsis* resistance to *B. cinerea* by enhancing the expression of *PDF1.2a*, *PR-3*, and *PR-4* (Catinot et al., 2015). Over-expression of *AtERF1*, *AtERF5*, *AtERF6*, and *ORA59* increased *Arabidopsis* resistance against *B. cinerea* by promoting the expression of *AtPDF1.2* (Lorenzo et al., 2003; Zarei et al., 2011; Son et al., 2012; Moffat et al., 2012; Cheng et al., 2013). Over-expression of *GmERF3* in transgenic tobacco enhanced resistance against tobacco mosaic virus (TMV) and activating the expression of *PR1*, *PR2*, and *PR4* (Zhang et al., 2009). Over-expression of *GmERF113* increased soybean resistance to *P. sojae* and increased transcript levels of *GmPR1* and *GmPR10-1* (Zhao et al., 2017). Our previous research demonstrated that *ZmERF105* over-expression lines enhanced the expression of several PR genes, including *ZmPR1a*, *ZmPR2*, *ZmPR5*, *ZmPR10.1* and *ZmPR10.2* after infection with *E*. *turcicum*, while the expression levels of these PR genes were reduced in *erf105* mutant lines (**Figure 8**). We speculated that *ZmERF105* may enhance maize defense against *E. turcicum* by directly or indirectly regulating these PR genes.

POD and SOD are the most important antioxidant enzymes to help eliminate the excessive accumulation of ROS in plants, to induce resistance or repair the damage (Liu et al., 2016). In the present study, the activities of SOD and POD in the *ZmERF105* over-expression lines were markedly higher than in the WT after infection with *E*. *turcicum*, and were compromised in the *erf105* mutant lines (**Figure 7**). These results demonstrated that *ZmERF105* can strengthen ROS scavenging capability to provide sufficient protection against oxidative damage.

The phytohormones ET, JA, SA, and ABA are important for plants to distinguish and resist distinctive pathogens (Pieterse et al., 2012; Van der Does et al., 2013). Over-expression of *AtERF5* resulted in increased resistance to *B. cinerea* through MeJA/ET signaling pathway, while showed increased susceptibility to the hemibiotroph *Pst* DC3000 *via* suppressing SA-mediated signaling pathway (Son et al., 2012; Moffat et al., 2012). *AtERF1* and *ORA59* are more resistant to *B*. *cinerea via* MeJA/ET-mediated signaling pathway (Berrocal-Lobo et al., 2002; Pre et al., 2008). *AtERF15* involved in defense against *B. cinerea* and *Pst DC3000* through SA signaling pathway (Zhang et al., 2015). *ERF11* acted as a regulator of the SA-mediated signaling pathway to enhance the *Arabidopsis* defense response against *Pst DC3000*. The expression levels of SA-associated genes *ZmPR1a* and *ZmPR5* exhibited significant increases in the *ZmERF105* over-expression lines but were decreased in the *erf105* mutant lines after *E. turcicum* infection (**Figure 8**). Furthermore, the expression of *ZmERF105* was induced by SA treatment (**Figure 3C**) and SA induces resistance to *E. turcicum* infection in *erf105* mutant lines (**Supplementary Figure S1**). These results demonstrated that *ZmERF105* may involve in defense response to *E. turcicum* in the regulation of SA signaling pathways. In addition, we further demonstrated that *ZmERF105* may positively regulate the expression of MeJA-associated gene and ET-associated gene in response to *E. turcicum*, and negatively regulate the expression of ABA-associated gene **Supplementary Figure S2**). The results showed that *ZmERF105* may act positively as regulators of MeJA and ET signaling pathways in resistance to *E. turcicum*, and negatively in ABA signaling pathway.

In conclusion, *ZmERF105* is a novel ERF gene and plays an important role in the plant defense system. ZmERF105 protein was exclusively localized to the nucleus. ZmERF105 acts as a transcriptional activator that binds to GCC-box elements. *ZmERF105* expression responded to *E*. *turcicum* treatment. *ZmERF105* positively regulated plant resistance against *E. turcicum via* regulating the expression of defense-related genes and the activities of antioxidant enzymes. Overall, our study provides new information to dissect the poorly understood mechanism of *ZmERF105* in plant immune pathways.

## Data Availability Statement

All datasets generated for this study are included in the article/**Supplementary Material**.

## Author Contributions

Conceived and designed the experiments: LJ and WGY. Performed the experiments and drafted the manuscript: ZZ, YL, SL, HW, and WM. Analyzed the data: ZW, WY, JC, and XR. Contributed reagents/materials/analysis tools: LJ and WGY.

## Funding

This research was supported by Science and Technology Development Project of Jilin Province (20190301013NY), Doctor Foundation of Jilin Agricultural University (201801) and “13th Five-Year” Science and Technology Project of Education Department of Jilin Province (JJKH20180660KJ).

## Conflict of Interest

The authors declare that the research was conducted in the absence of any commercial or financial relationships that could be construed as a potential conflict of interest.
