# Supplementary material for: A Novel ERF Transcription Factor, ZmERF105, Positively Regulates Maize Resistance to Exserohilum turcicum
Source: Front Plant Sci. 2020 Jun 16;11:850. doi: 10.3389/fpls.2020.00850 (PMC7308562; doi:10.3389/fpls.2020.00850)
Supplement: Supplementary file 1 [file Image_1.pdf]

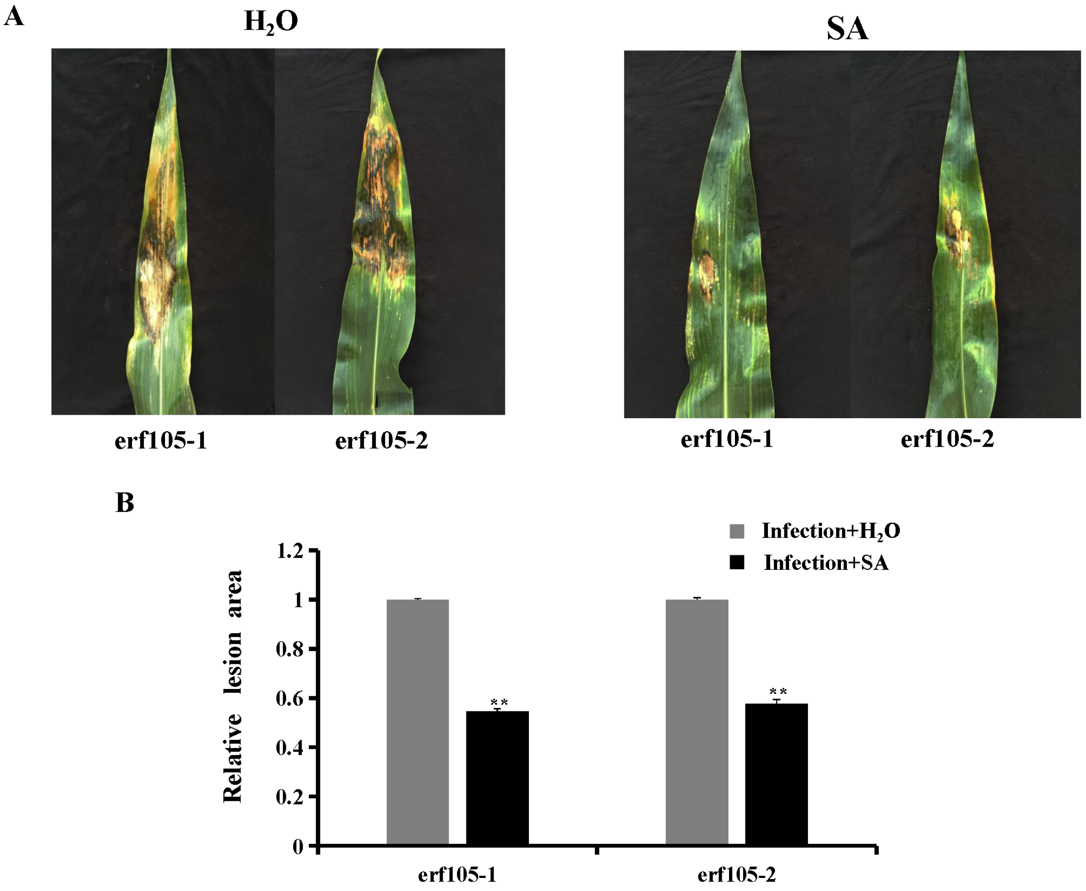

FIGURE S1 *ZmERF105* involved in SA-induced defense response. (A) SA induces resistance to *E. turcicum* infection in *erf105* mutant lines. Disease symptoms on the leaves of *erf105* mutant lines at 5 dpi. (B) The relative lesion areas of the *erf105* mutant lines were detected at 5 dpi. The experiment was performed using three biological and technical replicates each, and analyzed using Student's t-tests (**P<0.01). Bars indicate standard error of the mean (SE).
