# Supplementary material for: A Novel ERF Transcription Factor, ZmERF105, Positively Regulates Maize Resistance to Exserohilum turcicum
Source: Front Plant Sci. 2020 Jun 16;11:850. doi: 10.3389/fpls.2020.00850 (PMC7308562; doi:10.3389/fpls.2020.00850)
Supplement: Supplementary file 2 [file Image_2.pdf]

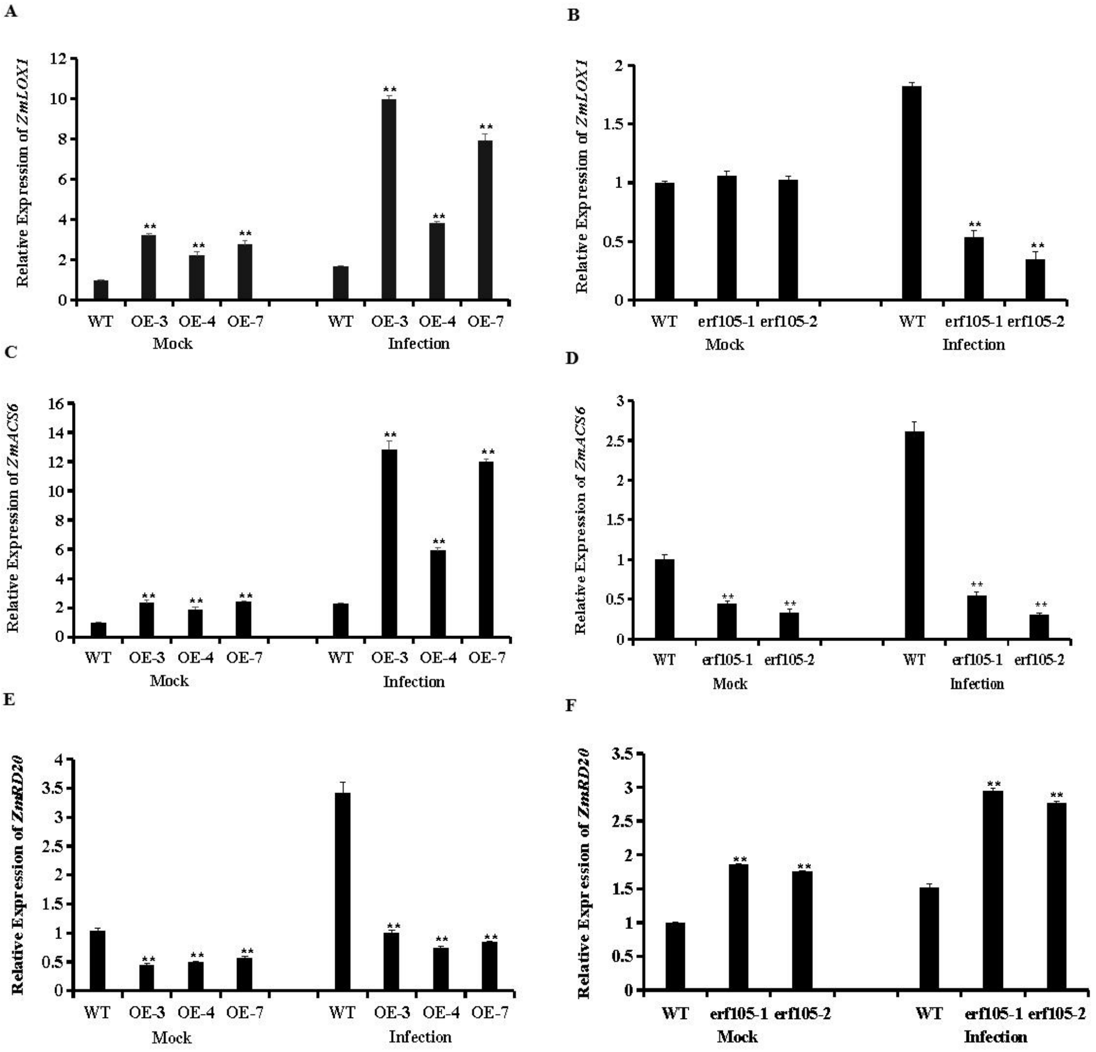

FIGURE S2 Relative expression levels of MeJA-related gene, ET-related gene ABA-related gene under mock treatment and infected by *E. turcicum* at 24 hpi in *ZmERF105* over-expression and *erf105* mutant lines, respectively. (A) and (B) The expression of the MeJA-related gene *ZmLox1*. (C) and (D) The expression of the ET-related gene *ZmACS6*. (E) and (F) The expression of the ABA-related gene *ZmRD20*. The relative expression levels were calculated by the $2^{-\Delta\Delta Ct}$ method with the maize housekeeping gene *ZmTub* (GRMZM2G066191) as an internal control. The expression of the control sample [mock-treated wild-type (WT) plants] was set to unity. The experiment was performed using three biological and technical replicates each and analyzed using Student's *t*-tests (**P < 0.01). Bars indicate standard error of the mean (SE).
