# Supplementary material for: A Novel ERF Transcription Factor, ZmERF105, Positively Regulates Maize Resistance to Exserohilum turcicum
Source: Front Plant Sci. 2020 Jun 16;11:850. doi: 10.3389/fpls.2020.00850 (PMC7308562; doi:10.3389/fpls.2020.00850)
Supplement: Supplementary file 3 [file Table_1.docx]

**Table S1. The primer sequences used in this study.**

| Cloning of *ZmERF105* | *ZmERF105-*F | GAGGCTGCGAGCTAACACA |
| --- | --- | --- |
|  | *ZmERF105*-R | TCCATAACTAGAAACCGCTAACAA |
| Overexpression | *ZmERF105*-3301-F | CCCATGGACTTCAGCGGTG |
|  | *ZmERF105*-3301-R | CCCACGTGACCGATGTCCCCA |
| Yeast | pGADT7-*ZmERF105*-F | CCCATGGACTTCAGCGGTG |
| one-hybrid | pGADT7-*ZmERF105*-R | CGGGATCCACCGATGTCCCC A |
|  | *ZmTub*-QF | CTACCTCACGGCATCTGCTATGT |
|  | *ZmTub*-QR | GTCACACACACTCGACTTCACG |
|  | *ZmERF105*-QF | CTAGCCTCCCCATCGTTACTA |
|  | *ZmERF105*-QR | CGTTCGTCAAGTCCACGTA |
|  | *ZmPR1a*-QF | GGCGAGAGCCCCTACTAGAC |
|  | *ZmPR1a*-QR | AAATCGCCTGCATGGTTTTA |
|  | *ZmPR2*-QF | ACGTTCTCATCCCACGACA |
|  | *ZmPR2*-QR | CTCGTCTACTAGGGTGGTGTGTT |
|  | *ZmPR5*-QF | GTCATCGACGGCTACAACCT |
| qRT-PCR | *ZmPR5*-QR | CACGGGCAGAAGGTGACT |
|  | *ZmPR10.1*-QF | AGATCACTAAAGCCAAGGAGTC |
|  | *ZmPR10.1*-QF | CATGGTCTAGTTGTAGGCTTCC |
|  | *ZmPR10.2*-QF | AGCCTTCAGCTAGCCCCAAGTT |
|  | *ZmPR10.2*-QR | GCGGAGGCCATTACTACTTCAG |
|  | *ZmACS6*-QF | TCATCACCAACCCTTCCAAC |
|  | *ZmACS6*-QR | AGTATATCTCGTCGCTCACCA |
|  | *ZmLox1*-QF | ACGTGAACGACTACTGCTGG |
|  | *ZmLox1*-QR | TCATGGTGTCACGGTAGTGC |
|  | *ZmRD20*-QF | CTCCTTTGCTGTCCATCCGT |
|  | *ZmRD20*-QR | TGAGCTCAACTCGTCTTTTGTT |
|  |  |  |
|  |  |  |
| Subcellular | pGADT7-*ZmERF105*-F | GGTACCATGGACTTCAGCGGTGAAATC |
| Localization | pGADT7-*ZmERF105*-R | TCTAGAGTTAACGGTGACCTCCGGGA |
